# Integrative multi-omics analysis identifies novel protein-coding genes and pathways in autism spectrum disorder: a comprehensive study

**DOI:** 10.1186/s12967-024-05642-5

**Published:** 2024-10-01

**Authors:** Deyang Liu, Yunxiang Liu, Xiaolu Fang

**Affiliations:** 1 Department of Rehabilitation Medicine, Xiangyang No.1 People’s Hospital, Hubei University of Medicine, Xiangyang, 441000 China; 2 Department of Clinical Laboratory, Xiangyang No.1 People’s Hospital, Hubei University of Medicine, Xiangyang, 441000 China

Autism Spectrum Disorder (ASD) is a complex neurodevelopmental condition characterized by a range of behavioral and communication challenges [1]. Understanding the genetic and molecular underpinnings of ASD is crucial for developing targeted interventions. This study employs an integrative analysis to reveal the significant genetic and molecular foundations of ASD, emphasizing the role of SLC30A9 in neuronal inhibition, endothelial cell maturation, and metabolism, thereby suggesting novel biomarkers and therapeutic targets for ASD.

Autism Spectrum Disorder data were sourced from the iPSYCH-PGC database, which included 18,381 ASD cases and 27,969 controls [2]. Proteomic data on 1475 plasma proteins were extracted from the dorsolateral prefrontal cortex of 376 participants [3], and validated with 198 additional samples [4]. Transcriptome data were obtained from GTEx V8 [5], and cell-type specificity data profiled from the CSEA-DB (https://bioinfo.uth.edu/CSEADB).Statistical analyses included several steps: MAGMA analysis assessed *P*-values of 18,841 genes using 1KGP LD information, with gene set analysis utilizing the MSigDB v.7.0 database, and forward selection identifying significant gene sets (*P* < 0.05). TWAS analysis used FUSION software to identify gene expression associations in the amygdala, with expression weights from GTEx V8 data. PWAS analysis computed SNP impacts on protein levels using predictive models (top1, blup, lasso, enet, bslmm), combined with GWAS z-scores using FUSION. Co-localization analysis employed the COLOC method to assess variant impact on ASD risk and protein levels with priors *p*1 = 10^-4, *p*2 = 10^-4, and *p*12 = 10^-5. An H4 value ≥ 0.75 indicated strong evidence for co-localization. MR analysis used SNPs with genome-wide significance (*P* < 5E-08) as instrumental variables to estimate causal effects. Cell-type specificity analysis used CSEA-DB data to map genetic signals to specific cell types. PPI networks were constructed using the GeneMANIA database for gene function analysis. LDSC and PheWAS explored ASD gene associations with other diseases. Single-cell RNA-seq analysis utilized the GSE165398 dataset from the hippocampus of ASD mice. Quality control was performed using the Seurat package, filtering cells with nFeature < 200 and mitochondrial/ribosomal gene expression > 10%. Cell annotation was done using SingleR and cross-verification. Differential expression of gene sets and hub genes was analyzed using Seurat’s AddModuleScore and the Wilcoxon test. Pathway differences were analyzed with the irGSEA package, pseudotemporal analysis was conducted using monocle2; and cell communication was investigated using the CellChat package. See Figure S1 for the study’s flowchart. The software versions and parameter settings used are provided in the Supplementary Material 4.

In our study, we used MAGMA to analyze ASD summary data, identifying 1,782 genes significantly associated with ASD and discovering 10 pathways related to its pathogenesis (Tables S1 and S2). Functional enrichment analysis highlighted pathways such as dorsal-ventral axis specification and T-tubule formation. Using the FUSION TWAS pipeline, we conducted a summary-based TWAS analysis, identifying 218 genes significantly associated with ASD (*P* < 0.05), with 65 validated by MAGMA (Fig. 1A). Additionally, PWAS confirmed the involvement of GSTZ1, MPI, and SLC30A9, whose cis-regulated brain and blood protein levels were linked to ASD (Figures S2A and Fig. 1A). Co-localization (COLOC) and Mendelian Randomization (MR) analyses provided robust evidence for the association of four proteins, particularly SLC30A9, with ASD, suggesting its potential impact on relevant biological processes (Figure S2B). Cell-type specificity analysis revealed a higher abundance of SLC30A9 in the brain, primarily involved in neuronal inhibition (Fig. 1B1 and 1B2). Protein-protein interaction (PPI) networks linked SLC30A9 to essential metabolic processes, including zinc ion homeostasis and response to metal ions (Fig. 1C). LDSC analysis showed ASD’s correlation with other mental disorders, such as depression, ADHD, schizophrenia, and loneliness (Table S4). PheWAS indicated a strong association between SLC30A9 and depression (Fig. 1D).

**Fig. 1:**
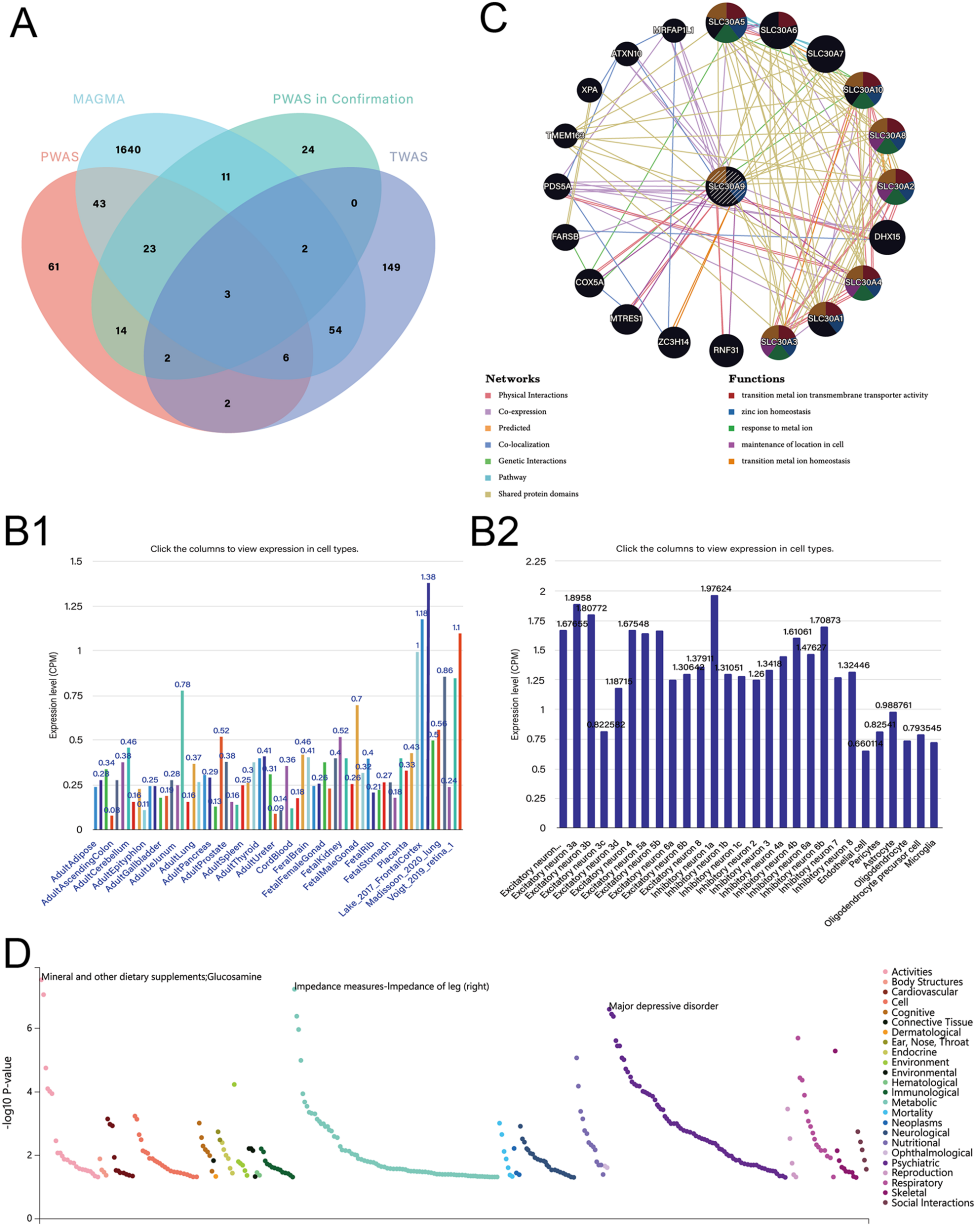
(**A**) Intersection of MAGMA, TWAS, PWAS in this study; (**B**) SLC30A9 is expressed in CSEA-DB database: (**B1**) SLC30A9 is expressed in bodily tissues; (**B2**) SLC30A9 is expressed in Brain cells. (**C**) Protein-protein interaction networks identified in this study. (**D**):PheWAS results in this study

Following stringent quality control measures, we analyzed 28,702 ASD cells and 13,576 control cells using SingleR and manual refinements to annotate hippocampal cell populations (Fig. 2A). Violin plots displayed distinct cell marker expression profiles between groups (Fig. 2B). Significant differences were observed in SLC30A9-related genes (Fig. 2C) and SLC30A9 expression itself (Fig. 2D), with a notable distribution of SLC30A9 expression at the cellular level (Fig. 2G). Notably, differences in endothelial cells were observed (Fig. 2E and F). Endothelial cells were categorized into High and Low SLC30A9 groups based on median expression values (Fig. 2H). Pathway analysis using Rank-based Reduction Analysis(RRA) showed increased activation of apoptosis, adipogenesis, and androgen response in high SLC30A9 cells (Fig. 2I). Pseudotemporal analysis revealed that higher SLC30A9 expression correlated with terminal differentiation states, implicating SLC30A9 in endothelial maturation in ASD (Fig. 2J and K, and 2L). Intercellular communication analysis indicated reduced interactions in ASD, except for increased signaling from neurons to fibroblasts and astrocytes to fibroblasts (Fig. 2M and N). The APP pathway was notably enriched in ASD, especially in interactions from endothelial cells to macrophages, suggesting a crucial role in ASD pathogenesis (Fig. 2O, P and Q). These findings highlight SLC30A9’s potential influence on endothelial cell behavior and intercellular signaling in ASD, providing new insights into the disease mechanisms.

**Fig. 2:**
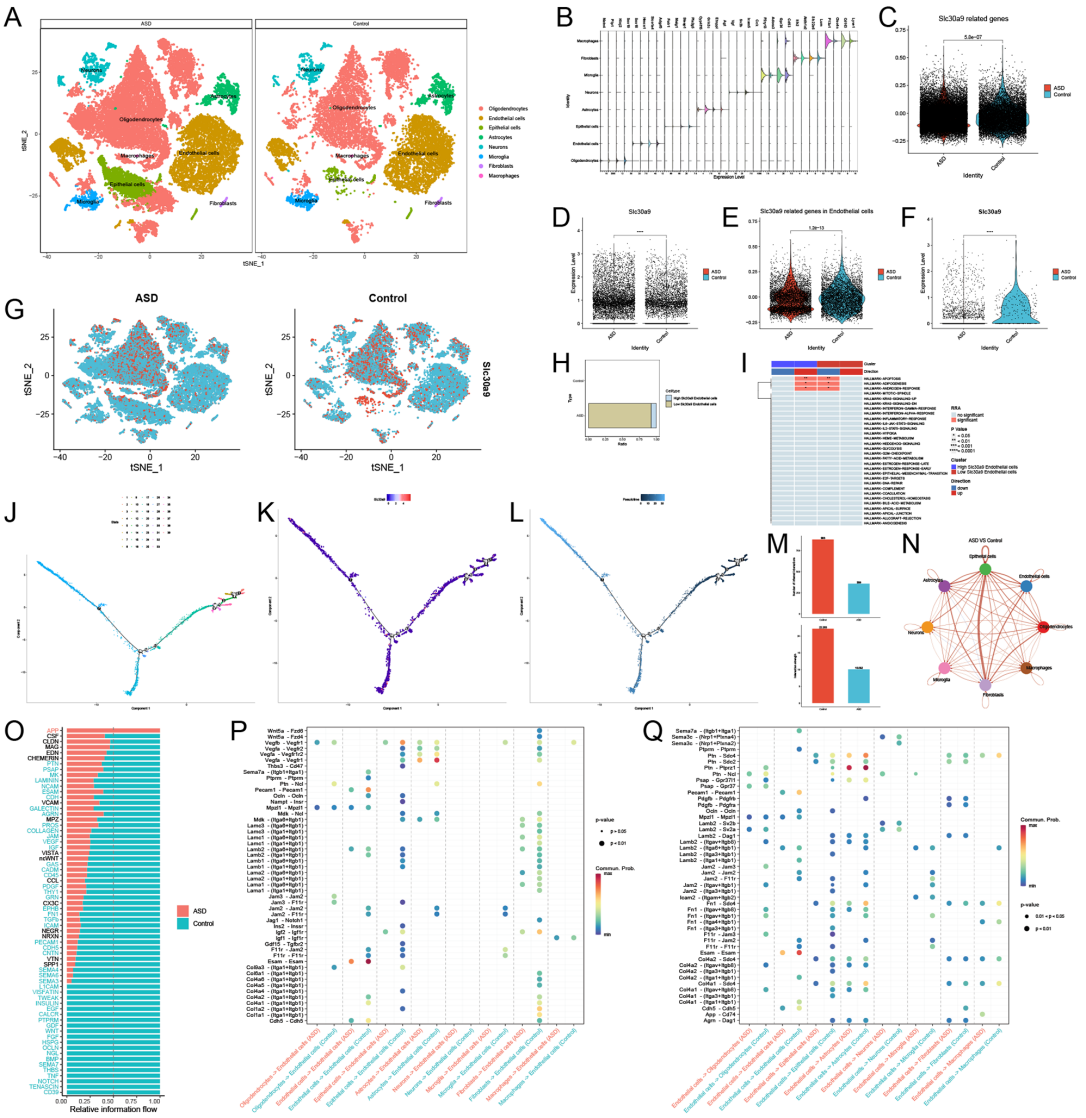
Single-cell analysis. (**A**) Cell annotation; (**B**) Violin plots of cell marker expression; (**C**) Difference in SLC30A9 related gene scores between two groups; (**D**) Difference in SLC30A9 expression between two groups; (**E**) Difference in SLC30A9 related gene scores in endothelial cells between two groups; (**F**) Difference in SLC30A9 expression in endothelial cells between two groups; (**G**) Distribution of SLC30A9 expression in cells of both groups; (**H**) Classification of endothelial cells based on high or low SLC30A9 expression; (**I**) Analysis of differences in HALLMARK signaling pathways between high and low groups; (**J**) Pseudotemporal state staging; (**K**) Distribution of SLC30A9 expression in pseudotemporal order; (**L**) Pseudotemporal time series distribution; (**M**) Histogram of interaction numbers and intensities between cells of both groups; (**N**) Differences in cell functional interactions between two groups; (**O**) Overall distribution of signaling pathways in both groups; (**P**)Heatmap of significant signaling pathways among cells with endothelial cells as target cells; (**Q**) Heatmap of significant signaling pathways among cells with endothelial cells as source cells

## Electronic supplementary material

Below is the link to the electronic supplementary material.

Supplementary Material 1: **Figure S1** Flowchart of overall study design

Supplementary Material 2: **Figure S2** PWAS and COLOC result (A)Proteome-wide association study analysis in this study; (B)COLOC and Mendelian Randomization result in this study

Supplementary Material 3: **Table S1** Genes Significantly Associated with ASD Identified by MAGMA; **Table S2** Functional Pathways Enriched in ASD Pathogenesis; **Table S3** Genes Significantly Associated with ASD Identified by TWAS; **Table S4** Genetic Correlation Between ASD and Other Mental Disorders Identified by LDSC

Supplementary Material 4: The software versions and parameter settings

## Data Availability

All data supporting the findings of this study are included within the Article and Supplementary Files. The raw single-cell RNA sequencing data have been previously deposited in the Gene Expression Omnibus database with the accession number GSE165398.

## Abbreviations

ASD: Autism Spectrum Disorder
iPSYCH-PGC: The iPSYCH-PGC database
GTEx V8: The Genotype-Tissue Expression (GTEx) database, version 8
MAGMA: Multivariate Analysis of Geno-Pheno Associations
MSigDB v.7.0: Molecular Signatures Database, version 7.0
TWAS: Transcriptome-wide Association Studies
PWAS: Proteome-wide Association Studies
COLOC: Co-localization analysis
MR: Mendelian Randomization
CSEA-DB: Cell Type-Specific Expression Atlas-Database
PPI: Protein-Protein Interaction
LDSC: Linkage Disequilibrium Score Regression
PheWAS: Phenome-Wide Association Studies
RRA: Rank-based Reduction Analysis
RNASeq: RNA Sequence data
scRNA-seq: Single-cell RNA sequencing
