# Supplementary figures and images for: Integrative multi-omics analysis identifies novel protein-coding genes and pathways in autism spectrum disorder: a comprehensive study
Source: J Transl Med. 2024 Oct 1;22:882. doi: 10.1186/s12967-024-05642-5 (PMC11443877; doi:10.1186/s12967-024-05642-5)

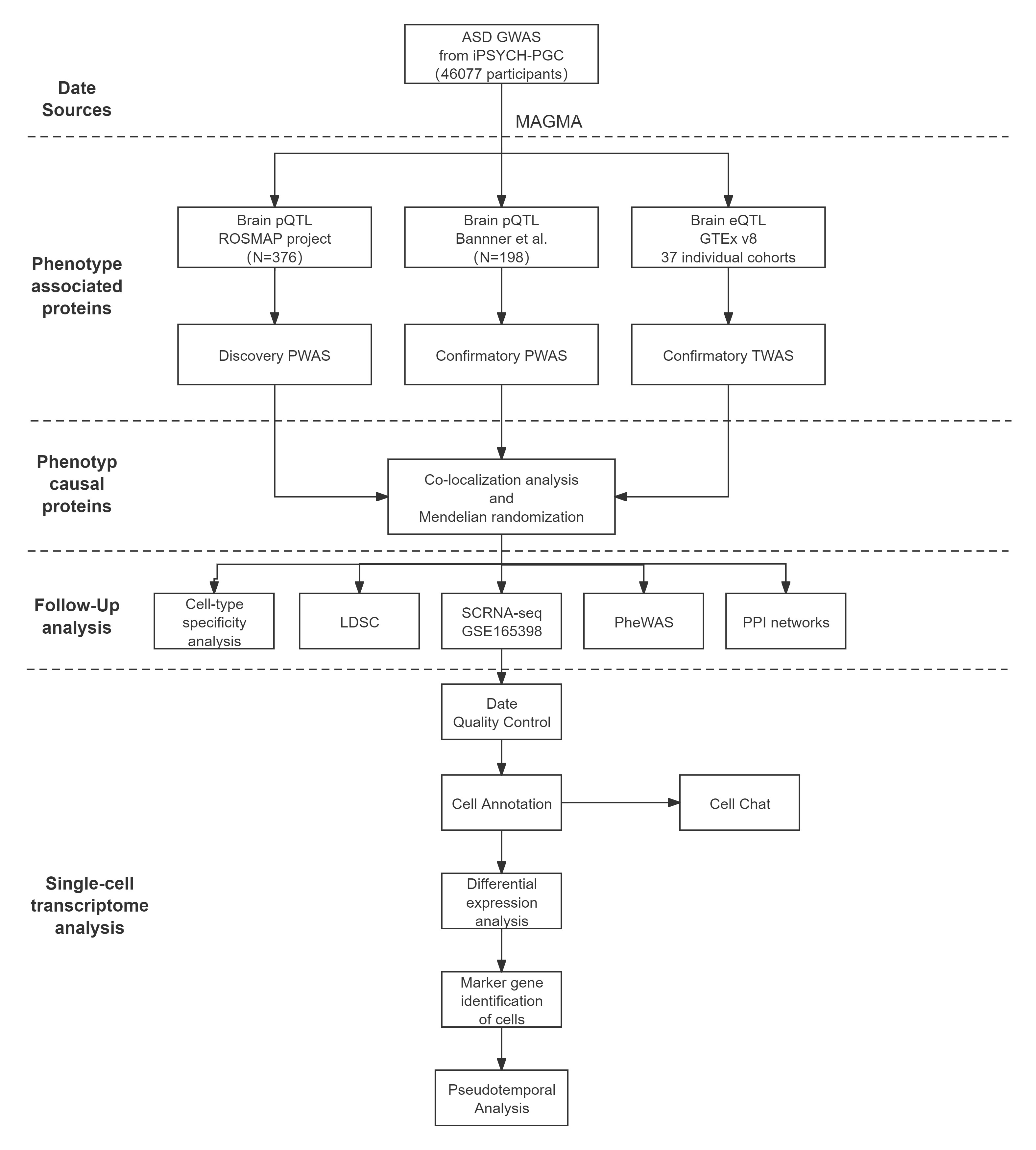

Supplement: Supplementary file 1 — Supplementary Material 1: Figure S1 Flowchart of overall study design [file 12967_2024_5642_MOESM1_ESM.jpg]

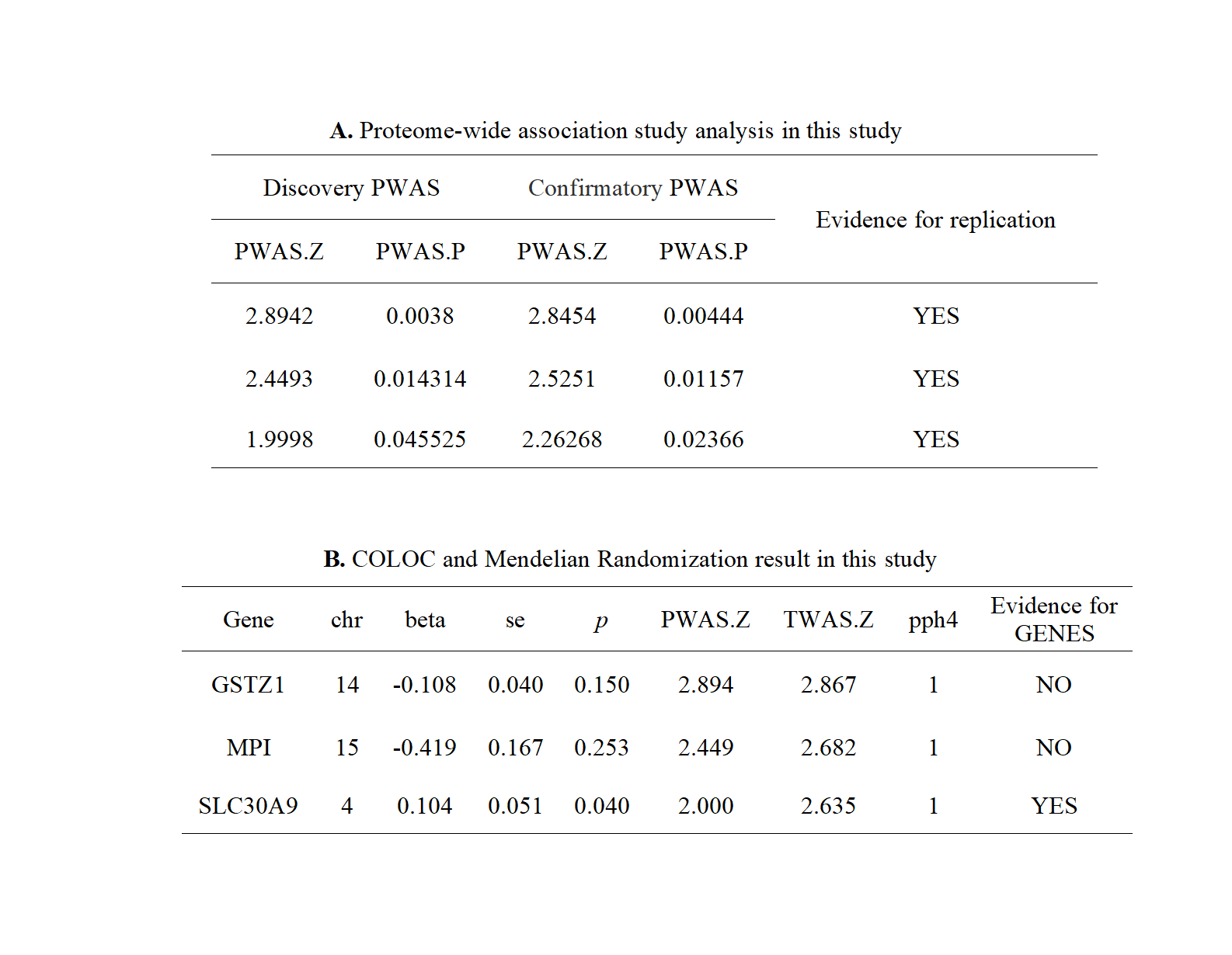

Supplement: Supplementary file 2 — Supplementary Material 2: Figure S2 PWAS and COLOC result (A)Proteome-wide association study analysis in this study; (B)COLOC and Mendelian Randomization result in this study [file 12967_2024_5642_MOESM2_ESM.tif]
