# Supplementary material for: Integrative multi-omics analysis identifies novel protein-coding genes and pathways in autism spectrum disorder: a comprehensive study
Source: J Transl Med. 2024 Oct 1;22:882. doi: 10.1186/s12967-024-05642-5 (PMC11443877; doi:10.1186/s12967-024-05642-5)
Supplement: Supplementary file 4 — Supplementary Material 4: The software versions and parameter settings [file 12967_2024_5642_MOESM4_ESM.docx]

The software versions and parameter settings.

1. MAGMA analysis: We used version v1.08. Default settings were applied for gene-analysis and gene-set-analysis.

2. TWAS analysis: FUSION software version v2.3 was employed, with default weights and models for gene expression association analysis.

3. PWAS analysis: FUSION models included top1, blup, lasso, enet, and bslmm, all conducted in an R v4.2.1 environment.

4. COLOC analysis: We used COLOC software version v4.0, with prior settings of p1=10^-4, p2=10^-4, p12=10^-5.

5. Single-cell RNA-seq analysis: Seurat package version v4.3.0 was used for quality control and analysis, monocle2 v2.20.0 for pseudotemporal analysis, and CellChat v1.1.3 for intercellular communication analysis.
